# Exsection of the Trunk of the Second Branch of the Fifth Pair of Nerves, beyond the Ganglion of Meckel, for Severe Neuralgia of the Face, with Three Cases

**Published:** 1859-04

**Authors:** J. M. Carnochan


### ARTICLE XVI.

*Exsection of the Trunk of the Second Branch of the Fifth Pair of Nerves, beyond the Ganglion of Meckel, for severe Neuralgia of the Face, with Three Cases.* By Prof. J. M. Carnochan.

The accounts of neuralgia of the face, or *tic douloureux,* heretofore given by authors, are of a very vague and indefinite character. Numerous essays and monographs have been written on this subject, since the time of Fothergill, who published, in 1776, an elaborate description of the disease, which attracted considerable attention. In all these efforts, the pathology of *tic douloureux* is described with ambiguity. In practice the treatment has been as empirical, as it has proved to be unsuccessful. The seat of the disease has been referred to distant irritations, especially in the splanchnic cavities—to a foreign body acting upon the nerve—to the pressure of bone upon some portion of the nervous trunks. By some authorities, it is referred to increased vascularity and thickening of the nerves ; while Astley Cooper, on the contrary, states, that the nerves present their natural color, and are rather diminished in size than enlarged. It can scarcely be supposed that beneficial result, should follow from treatment based upon theories so different in character.

*Tic douloureux* of the face, proper, or of the second branch of the fifth pair of nerves, is by far the most common form of facial neuralgia. This may be explained by the more numerous branches, which are given off by this trunk, and by the position which these branches occupy—in some places pent up in osseous canals, and in others subjected to exposures to changes in temperature, as well as to the agency of morbific influences, from which the other two trunks of the fifth pair are exempt.

The same laws which govern neuralgic disease of one of the branches of the fifth pair must be applicable to the disease in the other trunks. I believe that the phenomena of neuralgia can be explained with as much precision, as in any other disease which is well understood. In cases similar to those described below, whatever may have been the original exciting cause, I have no doubt that the real seat of the disease is in the trunk of the nerve, in front of the *foramen rotundum*—in some part of it, or in the whole of it. The causes of the disturbed and changed condition of the trunks of the nerve may be numerous—prolonged irritation upon the periphery—exposure—injuries—tumors—diseases of the teeth—pressure resulting from periosteal or osteal thickening of the osseous foramina or canals—sudden suppression of any of the important secretions, as of the catamenial discharge. From one or more of these causes, the trunk itself may be primarily affected, or, acting upon its ramifications, the irritation may be propagated to it. Prolonged irritation induces inflammation, and this generally remains passive or chronic. Some of the terminations of inflammation—such as the effusion of lymph among the interstices of the neurilemma or the nervous tissue itself—may become developed; leading to a vascular, engorged, thickened and enlarged condition of the nerve, or to a softening of it, at one or more points. In fact, vascular engorgement, or inflammation, with some of its consequences, of the neurilemma alone, or of it and the nerve together, by whatever cause produced, is the condition which constitutes the pathological changes in the trunk.

The three cases related below afford proof of what has just been stated. In each instance, the exsected nerve was found to be red, vascular, engorged, and considerably enlarged.

The diffused character of the pain can be easily understood, if we take into consideration the numerous ramifications of the second branch of the fifth pair, and the extensive surface over which their ultimate filaments are distributed. The periphery of the nerve occupies not only the superficial parts of the face, but extends deep amongst the bones of the upper jaw, to the nasal fossæ, to the septum nasi, to the hard and soft palate, to the pharynx, to the inner ear, to the orbit, and tc the temporal and malar regions.

It is well established, that if the trunk of a nerve be irritated along its course, the painful sensation will be referred to its periphery. If the ulnar nerve, for example, be struck where it passes behind the internal condyle, a sensation of pain is excited, which is referred to the little finger and to the ulnar border of the ring finger ; and if a prolonged irritation be kept up at this point, the skin of these fingers becomes tender to the touch, the sensibility being very much increased. The pain which is felt, at the knee, in *morbus coxarius*, also illustrates this law. "The obturator nerve," Sir Charles Bell remarks, "passes through the thyroid foramen, down to the hip joint, and, after supplying the muscles, is distributed upon the inner part of the knee. The nerve in its course is thus involved in the inflammation which affects the hip-joint, and the pain is referred to its extreme cutaneous branches, at a part distant from the seat of the disease."

It is by this principle—which governs the action of the stimuli upon the nerves of sensation—in connection with the anatomical distribution of the nervous ramifications, that the various phenomena of neuralgia can be explained. The disease being seated in the trunk of the nerve, we can readily understand that the pain must be referred to the peripheric extremities of the nerves, and will there be felt, as long as the branches are in communication with the encephalon.

From these views, we can see how futile the operation of division of the nerve at the *foramen infra-orbitale* must be. Where the trunk of the nerve is extensively diseased, no operation can rationally lead to a successful result unless all the branches emanating from the trunk are cut off from communication with the brain.

I believe that in such aggravated cases of neuralgia, the key of the operation is *the removal of the ganglion of Meckel, or its insulation from the encephalon.* Where even a large portion of the trunk of the second branch of the fifth pair has been simply exsected from the infra-orbital canal, the ganglion of Meckel continues to provide, to a great extent, the nervous ramifications, which will still maintain and keep up the diversified neuralgic pains. Besides the ganglion of Meckel, being composed of *gray matter, must play an important part as a generator of nervous power*, of which like a galvanic battery, it affords a continual supply; while the branches of the ganglion, under the influence of the diseased trunk, serve as conductors of the accumulated morbid nervous sensibility.

CASE I.—Henry Rousset, a French physician, residing in Greensborough, Caroline county, Maryland, consulted me in the early part of October, 1856, for severe neuralgia, which has for several years rendered him incapable of following his profession. He was of nervous temperament, good constitution, and sixty-nine years of age.

The disease first made its appearance in September, 1851, commencing with severe lancinating pains about the region of the left cheek and orbit. These pains continued for five or six days, and then disappeared, leaving him almost free from them for about four months. At the expiration of that time, the neuralgic pains again returned, with more violence, extending over the region of the left cheek, and continuing almost without intermission, for more than a week. After this exacerbation, the patient again became comparatively free from pain for a short interval; after which, the attacks returned with increased severity, and were renewed with greater frequency, more especially in the cold season, and in damp weather. As the disease progressed, the pain was not confined alone to the eye and cheek, but would also attack the lip and nose; each paroxysm being of longer duration than the preceding. With but slight variation, the disease went on in this way to harass and distress the patient for four years. About the commencement of March, 1856, the neuralgic exacerbations assumed a more violent form, marked by excruciating and almost unremitting suffering. He was, at this time, unable to eat, drink, converse, or laugh, without having a most violent paroxysm, causing him to shriek in anguish. The paroxysms were more severe during the night than day; sleep left him; his constitution began to give way, and his mind became much enfeebled. The slightest touch upon the surface of the face, a current of air or a mouthful of water acting on the palate, would throw a patient into a violent paroxysm of agony. During this long period of suffering, all the known remedies which have at times been extolled for neuralgia of the face had been tried—narcotics, tonics, anti-spasmodics, with counter-irritants, and galvanism—without producing any appreciable result. In this distressed condition, the patient, wearied of existence, and unable any longer to endure a life so made up of excruciating torture, presented himself to me for my advice, at the beginning of October, 1856. He expressed himself willing to undergo any operation, however severe, which held out the prospect of relief. Having no internal remedy to propose which had not already been administered, and having no faith in the mere division of the nerve upon the face, I proposed to him the exsection of the trunk of the second branch of the fifth pair of nerves to a point beyond the ganglion of Meckel. Being a physician himself, I explained at length my views, (as expressed above,) in regard to this malady. He immediately consented to have the operation performed, and desired that a near time should be appointed. I consequently agreed to perform the operation the following day, the 16th of October.

*Operation.*—The principal instruments necessary for this operation are a trephine, the crown of which is three quarters of an inch in diameter, an elevator, chisels of different shapes and sizes, a leaden or iron mallet, the bone forceps of Lüer, small pieces of sponge tied to a stick or a piece of whalebone, and a small fixed trephine of half an inch in diameter, which may be used to perforate the posterior wall of the antrum. The assistants being properly arranged, the patient was seated upon a solid chair, opposite a good light, and was put under the influence of chloroform. The head was rested upon the breast of an assistant, who maintained it in position. An incision was now made on the cheek, commencing near the internal angle of the eye, on the inferior edge of the orbit, opposite the anterior lip of the lachrymal groove. This incision was carried downwards and slightly outwards, for about an inch, to a point opposite the furrow on the lower portion of the ala of the nose; another incision, terminating at the same point, commenced about half an inch below the external angle of the eye, opposite the edge of the orbit; thus forming a V incision, in the area of which is situated the *foramen infra-orbitale.* The flap thus resulting was thrown upwards, and the branches of the second branch of the fifth pair of nerves sought for: some of these being found, they served as a ready guide to the trunk of the nerve. This was now insulated from the surrounding tissues up to the point of exit upon the face from the foramen. The lip was now everted, and the mucous membrane detached from the superior maxilla, along the line of junction between the cheek and the gum. A sharp-pointed bistoury was now inserted into the mouth, at the apex of the V incision, and carried downwards, so as to divide entirely the tissues of the cheek and upper lip, along a line passing midway between the ala of the nose and the commissure of the lips. The two flaps, thus formed, were now dissected from the osseous tissue beneath; one being reflected outwards, towards the ear, the other internally, towards the nose. The whole front wall of the *antrum maxillare*, with the nerve passing through the *foramen infra-orbitale*, was thus exposed. The crown of the trephine was now applied on the anterior wall of the antrum, immediately below the *foramen infra-orbitale*, and an irregular disk of bone removed, so as to expose freely the cavity of the antrum. The circumference of the foramen, the hardest portion of the canalis infra-orbitalis, was now destroyed by Lüer's forceps, and a small chisel. The trunk of the nerve was now traced along the osseous canal in the floor of the orbit, which was broken down with care, so as not to encroach upon the tissues in the cavity of the orbit. Arriving at the back of the antrum, the posterior wall of this cavity was broken down with a small chisel, and the portions of bone removed. The trunk of the nerve was now still further insulated from the other tissues in the *spheno-maxillary fossa*. The posterior dental nerves being divided, and the dissection being carried still further, the branches given off to form the ganglion of Meckel were reached. These were divided, and also the branch given off to run up towards the orbit. Lastly, by the use of blunt pointed scissors, curved on the flat side, the trunk of the nerve was divided from below upwards, close up to the *foramen rotundum*. The hemorrhage was not very profuse, the labial arteries being easily controlled by pressure of the fingers, and the branches of the internal maxillary artery, in the *spheno-maxillary fossa*, by dry lint, or, what is better, the compressed sponge. The lips of the wound were brought together and maintained in place by thirteen points of twisted suture, the German or Carlsbad pins being used.

This severe and trying operation is perfectly justified by the fearful nature of the disease for which it was projected. It is one of these operations which could not be supported by the patient without the influence of chloroform. The handling of so large a nervous trunk with the forceps, and the necessary contact with the hard instruments, while separating it from its surrounding connections, would, I suppose, be beyond human endurance, without the aid of the anæsthetic influence of chloroform or ether. For the rest, the effects of the cicatrices upon the countenance can scarcely be called disfiguring, and the patient speedily recovers, without suffering from much constitutional disturbance.

In this operation, and in those connected with the two succeeding cases, I was assisted by my colleague, Prof. Cox, by Drs. Proudfoot, Abrahams, Selden, Guleke, and Casseday: and by my pupils, Messrs. Dougherty, Scudder, and others.

*Condition of the Nerve.*—The trunk of the nerve, in this case, was much larger than natural, in nearly its whole extent. The neurilemma was very vascular, and the nervous tissue proper was all engorged in red: the trunk, after its removal, was so red as to have somewhat the appearance of muscular tissue. The length of the nerve removed was a little more than an inch and three-quarters. The lining membrane of the antrum was sound, as well also as the bones of the antrum and the osseous wall of the *canalis infra-orbitalis*.

*Progress of Union and After-Treatment.*—Oct. 16th. Six hours after the operation, the patient was visited. His pulse was 100; there was a slight fever; he complained of thirst, and lemonade was ordered. He spoke of a desire he had to vomit, which he ascribed to the chloroform. He stated that he felt slight twitchings on the nose, and at the corner of the lip.

17th (Friday.) The patient was remarkably well under the circumstances; sitting up; pulse 90; tongue lightly covered with a white fur; complained of pain in the wound; also of shooting pains in the left eye; he remarked that he could stick a pin into the upper lip and cheek without causing pain, there being no sensation in that region. Ordered chicken broth, and wine and water.

18th (Saturday.) Patient improving; wound healing; pulse natural; no fever; spoke of the numbed sensation in his face.

19th (Sunday.) Pulse full and natural; good appetite; partook of a beefsteak; in the afternoon four suture pins removed; slight pain in the wound; no return whatever of the neuralgia.

20th (Monday.) Cure progressing; healthy suppuration from wound; appetite excellent; general health much improved.

(Tuesday, Wednesday, Thursday.) During these days the rest of the pins were removed; patient felt no pain whatever either in the wound or cheek; wound in the antrum syringed with tepid water.

25th (Sunday.) Patient attended church; felt no pain whatever; incision of the upper lip and cheek entirely healed.

28th. Patient entirely well.

30th. Returned home to Maryland in high spirits, and delighted at the result of the operation.

December 7th, 1857. Fourteen months after the operation he writes to me that he is enjoying excellent health, and has been entirely free from neuralgic pain.

Case II.—Florence Cordello, a native of Italy, aged 54 years, of lymphatic temperament, chocolate maker by trade, was admitted to the State Hospital on the 14th of September, 1857, suffering from severe *tic-douloureux* on the left side of the face. The following is the account handed to me by the Assistant Surgeon, Dr. Guleke. In the year 1828, the patient contracted a very severe cold from exposure, and about this time he was seized with the pain for the first time.—According to his own description, the pain started from the *foramen infra-orbitale*, extending upwards to the forehead, and downwards into the teeth ; the paroxysm lasting about ten minutes. He supposed it to be tooth-ache, and had one or two teeth extracted. An interval of eight years took place, when he was again attacked with neuralgic paroxysms, lasting from five to ten minutes. Again, after the lapse of a year, the paroxysms reappeared in a more severe form, and at shorter intervals.

The patient, still believing his teeth to be the source of the disease, had all of them extracted on the left side of the upper jaw, but without any benefit. During these attacks he had been subjected to many kinds of treatment, both internally and externally : he also repaired to some of the mineral springs on the Rhine, but still to no purpose. He continued thus to suffer more or less intensely from the neuralgic paroxysms, for a period of time extending from 1837 to 1846, and with detriment to his general health. In 1846 while passing through the city of Heidelberg, in Germany, he consulted the celebrated Chelius with the hope of obtaining some beneficial result from his advice. That professor divided the nerve as it emanated from the *infra-orbital foramen*, by incisions from the mouth; and, six weeks after, again performed the same operation, without any favorable result. During the next six years the patient continued to suffer from neuralgic paroxysms of more or less intensity.

Oppressed by extreme suffering, he again sought relief from an operation, and in 1852 the nerve was again divided from the mouth, by forcing up the lip; the actual cautery being at the same time applied, by pushing the instrument from the mouth upwards into the wound, as far as the *foramen infra-orbitale*. This operation appeared to give some relief, and during the two succeeding years the patient's sufferings were somewhat alleviated. About two years ago, the paroxysms returned in the most aggravated form, progressed, and continued without much abatement. On the 1st of September last, being in New York, he again submitted to an operation for division of the nerve. This time, the branches of the nerve were divided by cutting through the integuments, directly upon the infra-orbital foramen; an operation causing no other effect than insensibility to the touch in the soft tissues near the infra-orbital foramen. Two weeks after this, he entered the State Hospital. The condition of the patient was then as follows: Notwithstanding the repeated division of the nerve, there was sensibility to the touch over the whole region of the cheek; the inner side of the lip alone appearing to be insensible. The patient describes the pain as starting from the *foramen infra-orbitale*, extending up as far as the *ligamentum palpebræ internum*, and also to the external corner of the eye; from the latter point, the pains shot down in nearly a straight line to a point about one inch to the outside of the left corner of the mouth, and a little below a line drawn horizontally on a level with the commissure of the lips. The pains, also, extended backwards, through the more deeply-seated portions of the face, shooting from the inner corner of the eye, along the base of the nose, and striking backwards towards the *spheno-maxil-*

*lary fossa.* The pain was of the true neuralgic character, and so intense as to drive the patient into a condition verging on delirium. A slight touch on the cheek, the inside of the mouth, or on the hard or soft palate, swallowing, or speaking, excited almost instantaneously the paroxysms in their severest form.

*The Operation.*—The operation in this case was performed after the same manner as the preceding, and was modified only by the greater depth of the antrum and face. There was also more hemorrhage from the spheno-maxillary fossa; this was controlled by compressed sponge pressed into the fossa. Supposing the hemorrhage might return, the lips of the wound were brought together by adhesive plaster, one suture only being used. The other sutures were inserted the following day. The nerve was cut from above downwards. The ganglion of Meckel was drawn out, hanging to the trunk of the nerve.

*Progress of Union and After-treatment.*—Compressed sponge was applied in the deeper portion of the wound; the external surface was closed with one suture; an anodyne was ordered for the night.

Oct. 11 (Sunday.) Patient slept well during the night; pulse 76; no bleeding; five suture-pins applied; ordered an anodyne.

12th. Patient slept well; no pain whatever; pulse 84; complained of thirst; but little appetite; spoke and swallowed without pain.

13th. Slept badly; had an attack of dysentery; pulse 96; felt a slight pulsating pain in the wound, which, however, was doing well; stated that there was no feeling over the surface of the left cheek, from the inner angle of the eye, descending along the nose to the lip, and upwards to the outer angle of the eye, including the lower lid; ordered opium and quinine. (*Afternoon,*) dysentery subdued; pulse 96; more cheerful.

14th. Patient improving; pulse 92; a number of the pins removed.

15th. Remaining pins removed; wound presented a healthy appearance ; pulse natural ; slight pain felt in the course of the wound.

16th. Removed the piece of compressed sponge, which had been placed at the back of the antrum, during the operation, to restrain the bleeding from the spheno-maxillary fossa.

18th. Patient doing well ; eat well, and slept naturally.

26th. Still entirely free from neuralgic pain ; the whole expression of the face changed from that of suffering and anxiety, to cheerfulness and serenity.

28th. Discharged from the hospital entirely cured, and in good health and spirits.

Dec. 8th. Visited the hospital ; still free from pain, and in good condition.

*Condition of the Nerve.*—The nerve in this case exhibited the same appearance as in the previous one. It was thickened, vascular, and engorged. The neurilemma and proper tissue of the nerve were both affected. The length of the trunk removed was two inches.

CASE III.—Mrs. Mary * * * * * a native of Portsmouth, England, and who had borne children, 55 years of age, of full habit and sanguineous temperament, consulted me, in the month of September, 1857, for severe neuralgia of the left side of the face. She had been a resident of the Northern States for thirty years, and had enjoyed, generally, remarkably good health.

On the 12th of August, 1851, while eating a plum in her garden, she was suddenly seized with a vivid shock of pain, commencing on her cheek, and passing through her jaw, as if caused by a sharp-pointed instrument, suddenly driven through her face ; shooting pains of this character, with intermissions of entire abatement, continued for sevaral days. A dentist was consulted, who, attributing the symptoms to the teeth, extracted several of them, but without the slightest benefit to the patient. The paroxysms continued, with more or less severity, for two months.

At the end of this time, they suddenly abated in their severity ; the respite lasting for about six weeks. Upon hearing of the sudden death of a friend to whom she was much attached, the paroxysms were again renewed, with greater frequency ; the intensity of pain increasing more and more with each succeeding attack. During the year 1852, the pain and paroxysms still continued with unyielding severity. The *tic* would now last for two and three months, with scarcely any of the intervals which had heretofore occurred. Cold air, the drinking of fluids, the slightest touch upon the cheek, or any sudden mental emotion, would invariably excite the most fearful paroxysms. During the year 1854, her condition was not in any way ameliorated ; the pain, if possible, was more severe, and her general health suffered from the want of rest. During the year 1855, the disease progressed with the same severity. In the early part of the year 1856, the paroxysms became still more aggravated ; the patient, at times, becoming almost delirious—starting up, running about her room, and screaming like a maniac. In the latter part of September, she sought relief from a surgeon in this city, who divided, by subcutaneous incision, the branches of the infra-orbital nerve, as it issues from the infra-orbital foramen.

About this time, she also took large quantities of various narcotics, and of the carbonate of iron. After the operation, she experienced some relief. The amelioration continued from October, 1856, until May, 1857, when the paroxysms were again renewed in their severest form.

The pain now became almost continual, depriving her nearly entirely of sleep ; she was unable to eat without torture, the act of swallowing invariably bringing on a paroxysm. During these exacerbations, the pain was diffused in different directions, extending from a point a little below the infra-orbital foramen, or from the ridge of the gums, and striking through the superior maxillary bone, towards the deeper portions of the face, and towards the orbit; and sometimes extending towards the region in front of the ear. She described the pain as of a beating character at times; each shock succeeding another in rapid succession, as if keeping time with the ticking of a clock. During this long period of suffering, she had been under the alternate care of several physicians; the various remedies most approved of in this kind of disease had all been faithfully and sedulously tried; stramonium, aconite, belladonna, hemlock, opium, morphia, chloroform, carbonate of iron, valerianate of ammonia, and other medicaments had been administered internally; while externally, in addition to the division of the nerve, blisters, sinapisms, hydrocyanic acid liniment, tincture of aconite, and chloroform had been resorted to—also electricity and galvanism. At the time I was consulted, she was suffering night and day from repeated and excruciating attacks, and, as she herself stated, she had visited the city to have an operation performed at all hazards, however desperate it might be, if I could only hold out any prospect whatever of its affording relief. Her general health was tolerably good, and she did not complain of loss of appetite. I explained to her the nature of the operation, which I believed to be the only one suited to her case. She immediately assented to submit to it as early as possible.

The *operation* was performed after the same precedure as heretofore described. The face was in this instance, also, very deep. The hemorrhage from the spheno-maxillary fossa was considerable, and was stopped by a piece of compressed sponge, to which a strong ligature was attached, by which it could be removed.

*Progress of Union and After-treatment.*—Nov. 5th (Thursday evening.) As soon as the operation was completed, the patient retired to her bed. Vomiting came on a few hours after, owing, probably, to the quantity of chloroform which had been used.

6th. Had slept tolerably well during the night; felt very little pain; pulse 80; no fever; complained of some pain in the wound, but had no neuralgic pain.

7th. Left side of the face slightly swollen ; puffiness about the eyelids ; has no pain ; has slept well without any anodyne ; states that she feels better than she has for months ; pulse 80 ; skin natural ; slight thirst ; five of the suture pins removed ; line of incision looks as though union by first intention was going on favorably. Still kept on fluids for nourishment—gruel, rice-water, ice-water, toast-water, and chicken tea. Ordered a gentle aperient.

8th. Had slept well ; tumefaction of face subsiding ; complains of headache ; cloth wetted with cold water applied on forehead ; same diet continued ; pulse natural ; removed the sponge which was used to stop the bleeding from the spheno-maxillary fossa ; this came away without any difficulty by slight traction, a little blood following. Complains of slight pain in the orbit. Removed six suture pins, leaving one only—that uniting the free border of the lip. Fluid diet as before.

9th. Patient slept well ; headache less ; pulse 78 ; no neuralgic pain ; a weak solution of the tincture of arnica ordered to bathe the cheek with ; removed the last pin ; union by first intention, along the line of incision, complete.

From the 9th until the 16th all has progressed favorably. No neuralgic pain whatever ; sleeps well ; swelling on the cheek diminished ; pain has entirely left the orbit ; secretion into the mouth from the wound in the antrum diminished. Ordered a gargle of the tincture of myrrh. Appetite has also returned. Had been sitting up, and walking about her room without any inconvenience. Has taken a little sulphate of magnesia ; has not required any anodyne.

Dec. 3d. The patient has been progressing favorably up to this time. The wound has healed entirely ; the line of cicatrix is becoming effaced ; not the slightest trace of tic douloureux remaining. There is no paralysis of the muscles of the face upon the side operated on.

In this case, the nerve was enlarged, very vascular, thickened and red. About two inches of the nerve were removed.

The bones of the cranium are liable to expansion, or thickening of their texture, from inflammatory action, most commonly dependent upon some constitutional taint. If the *os sphenoides* happened to be the seat of such disease, one or more of the foramina for the transmission of the nervous trunks might become very much contracted. A question might arise as to the effect of compression, from this cause, on the trunk of the second branch of the fifth pair, at the point where it is surrounded by the osseous sides of the *foramen rotundum*. From what has heretofore been stated, in relation to the law which governs the transmission of morbid sensibility along the trunk and branches of a nerve, subjected to an irritating cause, we should infer the supervention of neuralgia of the face, of the most severe character. In such a case, the operation of exsection of the trunk of the nerve, beyond the ganglion of Meckel, offers the best hope for relief; for, besides the removal of the trunk of the nerve, thus far, direct local depletion is obtained at the seat of the irritation; and, moreover, the portion of the nerve, placed in the foramen, will, most probably, become atrophied or diminished.

Pathological records corroborate the opinion which locates the seat of facial neuralgia on the nervous branches or trunks, after they have emerged from the encephalon.

After the section of the fifth pair of nerves, within the cranium, it is a well established fact that the *general sensibility* is annulled in the superficial and deep parts of the face; that their nutrition and their secretions are more or less perverted; and that the functions of the organs of *special sense* are disturbed. From this physiological fact, we arrive at the important diagnostic conclusion, that disease, involving the trunk of the fifth pair, and the ganglion of Gasser, so as to compromise its connections with the grand sympathetic, must be attended with pathological manifestations in the external organs of sense; the most remarkable of which are observed in the globe of the eye.

Cases illustrating this statement—important also in regard to the prognosis—are related by Herbert Mayo, Abercrombie, and others. The following case, published by M. Serres (Anatom. Comp. du cerveau, etc.,) is to the point.

"Adroite, l'insensibilité de la conjonctive était telle qu'on pouvait passer entre les paupières et le globe de l'œil les barbes d'une plume sans que le malade s'en aperçût ; il y avait immobilité complète du globe de l'œil et de ses dépendances ; la narine droite était également insensible à l'introduction d'un corps étranger ; toutefois l'odorat n'avait pas complétement disparu. Le malade ne recevait aucune impression de l'application du sulfate de quinine sur la moitié droite de sa langue. Les gencives du même côté étaient molles, fongueuses, noirâtres, détachées des os. Il y avait eu successivement inflammation de l'œil droit, coarctation de la pupille, opacité de la cornée et enfin perte de la vue. L'ouïe était diminuée à droite quelques jours avant la mort. *A l'ouverture du cadavre, on trouva la cinquième paire ramollie à son origine jaunâtre et presque gélatiniforme. Cette altération s'enfonçait à une ligne ou deux dans la protubérance annulaire. Le ganglion de Gasser, de ce côté était d'une ligne et demie plus large que du côté sain ; il était jaunâtre. Quant à la petite racine du trijumeau, elle était intacte.*"

